# Hydration status and fluid intake of urban, underprivileged South African male adolescent soccer players during training

**DOI:** 10.1186/s12970-015-0080-0

**Published:** 2015-05-03

**Authors:** Reno Eron Gordon, Susanna Maria Kassier, Chara Biggs

**Affiliations:** Discipline of Dietetics and Human Nutrition, University of KwaZulu-Natal, Private Bag X01, Scottsville, 3209 South Africa

**Keywords:** Hydration, Fluid, Adolescent, Soccer, Male

## Abstract

**Background:**

Poor hydration compromises performance and heightens the risk of heat stress which adolescents are particularly susceptible to as they produce comparatively larger amount of metabolic heat during exercise. This study determined the hydration status and fluid intake of socio-economically disadvantaged, male adolescent soccer players during training.

**Methods:**

A pilot study was conducted among 79 soccer players (mean age 15.9 ± 0.8 years; mean BMI 20.2 ± 2.1 kg/m^2^). Hydration status was determined before and after two training sessions, using both urine specific gravity and percent loss of body weight. The type and amount of fluid consumed was assessed during training. A self-administered questionnaire was used to determine the players’ knowledge regarding fluid and carbohydrate requirements for soccer training.

**Results:**

Players were at risk of developing heat illness during six of the 14 training sessions (60 - 90 minutes in length). Although on average players were slightly dehydrated (1.023 ± 0.006 g/ml) before and after (1.024 ± 0.007 g/ml) training, some were extremely dehydrated before (24%) and after (27%) training. Conversely some were extremely hyperhydrated before (3%) and after training (6%). The mean percent loss of body weight was 0.7 ± 0.7%.

The majority did not consume fluid during the first (57.0%) and second (70.9%) training sessions. An average of 216.0 ± 140.0 ml of fluid was consumed during both training sessions. The majority (41.8%) consumed water, while a few (5.1%) consumed pure fruit juice. More than 90% stated that water was the most appropriate fluid to consume before, during and after training. Very few (5.0%) correctly stated that carbohydrate should be consumed before, during and after training.

**Conclusions:**

Approximately a quarter were severely dehydrated. Many did not drink or drank insufficient amounts. The players’ beliefs regarding the importance of fluid and carbohydrate consumption did not correspond with their practices. A nutrition education programme is needed to educate players on the importance of fluid and carbohydrate to prevent dehydration and ensure appropriate carbohydrate intake.

## Background

It is important for athletes to maintain an optimum hydration status in order to prevent dehydration, and to support cardiovascular and thermoregulatory functions needed for optimum athletic performance [1]. Dehydration can result in a decrease in aerobic performance, an increase in core body temperature and a reduction in the athletes’ ability to tolerate heat produced during exercise, potentially compromising health and performance [2,3]. The prevention of dehydration may be of greater significance in the younger players as adolescents are at a greater risk of suffering from heat illness as they produce a greater amount of metabolic heat compared to adults, mainly due to their greater surface area to body weight ratio and they cannot produce sweat as efficiently as adults [4,5]. Children’s core body temperature increases more rapidly during dehydration and they have a higher sweating threshold compared to adults [5].

In adults, dehydration of 2% to 3% may reduce strength, decrease power output, impair physiologic function and exercise performance capacity although, no affect has been demonstrated on sprint performance [6]. As dehydration exceeds 3 to 5%, heat stress is a risk which reduces heat tolerance and work capacity [7,8]. At these levels sweat production and skin blood flow begins to diminish [9]. Plasma volume decreases due to dehydration resulting in an increase in submaximal heart rate, a reduction of cardiac output and a diminished capacity for heat dissipation [10]. If muscle blood flow continues to decrease due to dehydration, reduced nutrient delivery, diminished metabolite removal and changes in cellular metabolism may occur [10]. The effect is compounded if an athlete is dehydrated before a high-intensity exercise session as this can further increase the physiological strain and decrease performance [6].

Although as little as a 2 to 3% reduction in body mass has been shown to negatively impact the performance of adults, the extent of dehydration required to be deleterious to adolescent athletes is undetermined [8,11]. Children experience reduced endurance performance at 1% dehydration and may not hydrate adequately when consuming water during training, thereby predisposing them to dehydration [7,12]. Although adequate hydration is important for athletes, it is often ignored as an important aspect of both training and competitive matches [4,13,14].

Although there are a substantial number of publications on the hydration status and fluid intake of adolescent athletes in intermittent sports including soccer, this study is unique as the hydration practices of socio-economically disadvantaged adolescent soccer players in Africa has not been investigated. Soccer is the most popular sport in South Africa, especially among underprivileged adolescents, and is therefore regarded as a “black sport” or a sport of the poor [15,16]. However, South African adolescents face many challenges that include unemployment, poverty and food insecurity, all of which could compromise the implementation of appropriate sports hydration strategies further threatening their health and well-being. In Pietermaritzburg the capital city of KwaZulu-Natal (South Africa) where the study was conducted 43.1% of youths are unemployed and 16.1% of households reported having no income to sustain the household [17,18]. The aim was to establish baseline data by observing current practices in socio-economically disadvantaged communities to identify areas that require intervention.

## Methods

### Research participants

A pilot study was conducted to document the hydration practices of South African male adolescent soccer players. The study sample included 79 amateur 14 to 17 year old players from seven teams in the Pietermaritzburg and District Soccer Association (PADSA) league from March 2011 to August 2011. Players resided in socio-economically disadvantaged urban areas in and around Pietermaritzburg, the capital city of KwaZulu-Natal, a province in South Africa.

Ethical clearance was obtained from the Human and Social Sciences Ethics Committee of the University of KwaZulu-Natal (Protocol HSS/1297/2010 M). Participation was voluntary and the participants were given the option to withdraw at any time without being subject to any negative consequences. Written informed consent was obtained from each player prior to participation in the study and the parents/guardians were provided with a letter informing them of their son’s decision to participate in the study and what participation entailed.

### Testing sessions

Each team was assessed on two training sessions that were two days apart. Training sessions were held after school in the afternoon. An average training session with a duration of 60 to 90 minutes, included 20 minutes of stretching, sit ups and drills such as sprints and slalom followed by a game. Teams six and seven played games lasting 40 minutes this was because of the limited winter daylight time. On arrival at the first training session (TS1), players were weighed in minimal clothing (underwear) after producing a mid-stream urine sample. The scale (Masskot UC-300 capacity 50 g to 200 kg; accuracy 50 g) was placed on a stable level area and calibrated using a five kilogram calibration weight (Avery Weigh-Tronix) before each training session. Weight measurements were repeated twice and a third taken if the difference between the two measurements was greater than 100 g. The mean of the two closest measurements was used as the final weight.

The change in hydration status was measured by percent change in body weight using the following equation:

[(Pre-training body weight (kg) – Post-training body weight (kg)) / Pre-training body weight (kg)] × 100 [19].

Obtaining both the urine samples and body weight measurements was challenging as most training grounds did not have change rooms and ablution facilities as they were situated in poor socio-economic areas.

Hydration status was determined by urine specific gravity (U_SG_) measured with a portable digital urine refractometer (Atago Uricon UG-1 D20; range 1.000 to 1.080; accuracy ± 0.001). Each player was issued with a urine sample bottle labelled with their unique identification code. To obtain a mid-stream urine sample, players were instructed to pass a little urine before half filling the bottle and then replacing the lid. The sample bottle was returned to the primary investigator who placed them in a closed polystyrene box away from direct sunlight and subsequently measured the U_SG_ according to the manufacturer’s directions within an hour post collection. Each measurement was repeated twice. If the measurements differed by 0.001 g/ml, a third measurement was taken and an average of the two closest measurements was used.

The U_SG_ hydration classification for adults was used as there were no published reference values for adolescent athletes (Table 1). The adult participants on whom this classification is based were active but not highly trained and did not undertake intense physical training or exercise in a hot environment [20]. These conditions are similar to the participants in the current study who were also not highly trained and on average did not exercise in a hot environment.

**Table 1:** **Classification of hydration status using urine specific gravity**

**Hydration category**	**Urine specific gravity**
Extremely hyperhydrated	<1.012
Slightly hyperhydrated	1.012-1.014
Well hydrated	1.015-1.017
Euhydrated	1.018-1.020
Slightly dehydrated	1.021-1.024
Very dehydrated	1.025-1.027
Extremely dehydrated	>1.027

[20].

The players’ height (Seca Leicester stadiometer; range 20 to 207 cm) was measured on inhalation. Height measurements were repeated twice and a third taken if the difference between the two measurements was greater 0.2 cm. The mean of the two closest measurements was used as the final height.

Body mass index (BMI) was calculated using the following equation [21]:

BMI = body weight (kg) / height (m) ^2^. The age and BMI were plotted on the World Health Organisation (WHO) BMI-for-age Z-score charts for boys aged 5 to 19 years to determine the Z-scores. These Z-scores were then classified according to the WHO reference ranges (Table 2). A normal BMI was defined as a Z-score between -2 and 1.

**Table 2:** **Classification of BMI-for-age**

**Z-score**	**Classification**
Above 3	Obese
Above 2	Overweight
Above 1	Possible risk of overweight
0 (median)	Normal range
Below -1	Normal range
Below -2	Wasted
Below -3	Severely wasted

[26].

An automatic weather station, placed in the open approximately 5 m from the side touchline, recorded relative humidity (RH) and air temperature during the training sessions. The weather station consisted of a Vaisala and CS500 humidity/temperature sensor which were placed in a 6-plate radiation shield, allowing air to pass freely to the sensor keeping it at or near ambient temperature. For RH, the sensor had an operating range of 0 to 100% RH. In the 0 to 10% range, the accuracy of the sensor was not specified. In the 10 to 90% range the accuracy of the sensor was ± 3.0% while in the 90 to 100% range the accuracy of the sensor was ± 6.0%. For measuring temperature, the sensor had a measuring range of -40 to 60°C. Data was recorded every minute by a battery powered CR200 data logger using PC200W software. The weather station was piloted before data collection to determine if all the instruments were functional and if the weather station was collecting the necessary environmental data.

The heat index was calculated from the following equation where T represents the ambient temperature in degrees Fahrenheit (°F) and R represents the RH [22]:

Heat index = - 42.379 + 2.04901523 T + 10.14333127R - 0.22475541TR - 6.83783 × 10-3 T^2^ - 5.481717 × 10-2R^2^ + 1.22874 × 10-3T^2^R + 8.5282 × 10-4TR^2^ - 1.99 × 10-6T^2^R^2^

Table 3 shows the classification used to determine the risk of heat illness [23].

**Table 3:** **Classification of the risk of heat illness according to heat index**

**Heat index**		**Risk of heat illness**	**Possible effects**
**°F**	**°C**		
<80	<26.7	No risk	No risk for developing heat illness
80 to 89	26.7 to 31.7	Caution	Fatigue possible with continued exposure and physical activity
90 to 104	32.2 to 40	Extreme caution	Sunstroke, heat cramps, and heat exhaustion possible
105 to 129	40.6 to 53.9	Danger	Sunstroke, heat cramps, and heat exhaustion likely, and heat stroke possible
>130	>54.4	Extreme danger	Heat stroke highly likely with prolonged exposure

The training grounds were not in close proximity to schools. Therefore, the players made use of the notoriously unreliable public transport or walked and arrived just prior to training. As players needed to return home before night fall (17 h15 to 18 h00), they left immediately after training ended. It was therefore not possible to monitor fluid intake for 30 to 60 minutes before or after training. As their typical behaviour was being observed, the players did not receive any advice on hydration practices for training purposes. Since the teams surveyed had never received formal education on hydration or sports nutrition, it was assumed that the monitoring would not significantly alter their behaviour as the players would be unlikely have the knowledge on how to change it.

No beverages or refreshments were provided for any of the teams and none were available in the vicinity of the training grounds. In addition, no food was consumed during training. Players consuming fluids from drink bottles were instructed not to spit out the fluid consumed from the bottle, or pour it on their heads to cool down or throw any fluid away. The fluid intake during training was measured by determining the weight (Soehnle electronic scale; capacity 10 kg, accuracy 2 g) of the drink bottles before and after consumption. Four research assistants monitored the fluid consumption of players during training. As no players brought additional fluid, bottles were refilled at a tap only if there was one available at the training ground.

Three training grounds did not have taps and the remaining four only had one tap in the vicinity of the field. An additional two research assistants monitored fluid consumption from the tap. The weight of the bottle before and after refilling was recorded. Some players drank mouthfuls directly from the tap. They were not given cups to drink from to facilitate measurements as this may have biased their fluid intake and also were not typical practice. The number of mouthfuls consumed was recorded by the research assistants stationed at the tap. After training to calculate the volume of the average mouthful, players drinking from the tap were instructed to spit three mouthfuls into a measuring container and the mean volume was calculated as the sum of mouthful volumes / 3. To calculate total fluid intake the weight of the drink bottles during training was subtracted from the weight after training and the difference was the amount of fluid consumed, assuming that 1 g is equal to 1 ml of fluid. The fluid intake from drink bottles was added the volume from mouthfuls consumed, together this comprised total fluid intake. The type of fluid was also recorded.

At completion of training and before consuming any fluid, the players were towel dried and weighed in the same minimal clothing after producing a mid-stream urine sample. The same procedure was followed in the second training session (TS2) with the exception of measuring height.

Knowledge regarding the importance of carbohydrate and fluids, as well as the amount and type of carbohydrate and fluid required for optimal training, was measured at training session three using a self-administered questionnaire. The questionnaire consisted of 25 open and close ended questions divided into two sections. The first section assessed the players’ knowledge regarding the type and amount of fluid and when to consume this before, during and after training, while the second section evaluated the players’ knowledge regarding the importance of carbohydrate and when to consume this before, during and after training. The questionnaire, which had previously been used in several South African studies, was piloted before the study [24,25].

### Data analysis

Descriptive statistics were used to describe the characteristics of the players. The chi-square test was used to determine the difference between the number of players who lost or gained weight during training. The chi-square test was also used to determine the difference between the number of players in each U_SG_ category before and after training. The paired samples t test was used to determine the difference between training sessions for the percent change in body weight. The paired samples t test was also used to determine the difference in U_SG_ measurements between training sessions. Differences between variables were considered to be statistically significant if the p-value was < 0.05.

## Results

### Sample characteristics

A total of 79 soccer players participated in the study. Table 4 presents data on the sample characteristics. The majority (71%, 56/79) of the players were black. Although the mean BMI was normal (20.2 kg/m^2^, Z-score <1 to > -2), 28% (22/79) had a BMI-for-age Z-score less than -3 and were therefore classified as being severely wasted [26].

**Table 4:** **Sample characteristics**

**Sample characteristics**	
Mean age (years)	15.9 ± 0.8
**Race**	**n (%)**
Black	56 (71)
Mixed ancestry,	16 (20)
Indian,	7 (9)
**Anthropometry**	**Mean ±** ***SD***
Mean height (cm)	167.1 ± 5.7
Mean weight (kg)	56.6 ± 5.4
Mean BMI (kg/m^2^)	20.2 ± 2.1
**Nutritional status classified according to BMI-for-age (Z-score)**	**n (%)**
Obese (>3)	0 (0)
Overweight (>2 to 3)	0 (0)
Possible risk of overweight (>1 to 2)	4 (5)
Normal range (<1 to -2)	53 (67)
Wasted (< -2 to -3)	0 (0)
Severely wasted (< -3)	22 (28)

Nutritional status classified according to BMI-for-age was based on WHO Child Growth Standards [26].

Values for age, height and weight are means ± standard deviations.

Values in brackets are the number of players relevant to a sample characteristic over the total number of players.

### Urine specific gravity

Table 5 indicates the hydration status based on the U_SG_ measurements before and after training [20]. The mean U_SG_ was 1.023 g/ml (±0.005) before training and 1.024 g/ml (±0.006) after training, indicating a state of slight dehydration [20]. However, some players started (TS1 19%, TS2 28%) and ended (TS1 24%, TS2 29%) training extremely dehydrated. A few started (TS1 4%, TS2 3%) and ended (TS1 8%, TS2 5%) training extremely hyperhydrated [20].

**Table 5:** **Urine specific gravity before and after training**

**Variable**	**Training session 1**	**Difference between measurements before and after training (p-value)**	**Training Session 2**	**Difference between measurements before and after training (p-value)**	**Difference between the first and second training sessions (p-value)**
Mean U_SG_ before training (g/ml)	1.023 ± 0.005	0.145 *	1.023 ± 0.006	0.279 *	0.928 *
Mean U_SG_ after training (g/ml)	1.023 ± 0.007		1.024 ± 0.006		0.873 *
Hydration status before training, n (%)^҂^					
• Extremely hyperhydrated	3 (4)	0.184^#^	2 (3)	0.646^#^	0.692^#^
• Slightly hyperhydrated	3 (4)	0.692^#^	4 (5)	0.464^#^	0.573^#^
• Well hydrated	4 (5)	0.039^#^	8 (10)	0.419^#^	0.349^#^
• Euhydrated	9 (11)	0.790^#^	9 (11)	0.176^#^	0.320^#^
• Slightly dehydrated	24 (30)	0.733^#^	23 (29)	0.015^#^	0.592^#^
• Very dehydrated	21 (27)	0.256^#^	11 (14)	0.000^#^	0.000^#^
• Extremely dehydrated	15 (19)	0.363^#^	22 (28)	0.384^#^	0.604^#^
Hydration status after training n (%)^҂^					
• Extremely hyperhydrated	6 (8)		4 (5)		0.420^#^
• Slightly hyperhydrated	2 (3)		5 (6)		0.607^#^
• Well hydrated	1 (1)		3 (4)		0.780^#^
• Euhydrated	11 (14)		5 (6)		0.699^#^
• Slightly dehydrated	21 (27)		15 (19)		0.061^#^
• Very dehydrated	19 (24)		24 (30)		0.896^#^
• Extremely dehydrated	19 (24)		23 (29)		0.000^#^

*: Paired samples t test ^҂^: According to urine specific gravity measurements.

^#^: Chi-square test Urine specific gravity based on human hydration reference values developed for adults [16].

Significantly fewer players were well hydrated before (5%) and after (1%) the first training session (p = 0.039). For the second training session significantly fewer players (29% vs 19%) were slightly dehydrated after training (p = 0.015) while significantly more players (14% vs 30%) were very dehydrated after training (p < 0.001).

When comparing the difference between the two training sessions, there were significantly (p < 0.001) fewer players (TS1 27% vs TS2 14%) very dehydrated before the second training session than before the first training session. However, significantly (p < 0.001) more players were extremely dehydrated after the second training session than after the first training session (TS1 24% vs TS2 29%).

### Percentage change in body weight

Table 6 presents data on the change in body weight of players during the first and second training session. There was no significant difference in the amount of weight lost during the first training session (0.4 ± 0.3 kg) and the second training session (0.4 ± 0.4 kg)

**Table 6:** **Change in body weight**

**Variable**	**Training session 1 (n = 79)**	**Training session 2 (n = 79)**	**Difference between training sessions (p-value)**
Mean weight loss (kg)	0.4 ± 0.3	0.4 ± 0.4	0.720*
Percentage change in weight (%)	0.7 ± 0.5	0.7 ± 0.7	0.796*
Players who lost ≥ 2 % weight, n (%)	1 (1)	1 (1)	1.000^#^
Players who gained < 1% weight, n (%)	4 (5)	10 (12)	0.125^#^

*: Paired samples t test.

^#^: Chi-square test.

.

(p = 0.720), although during both training sessions one player lost more than or equal to 2% body weight while four to ten players gained less than 1% body weight.

### Team specific data

Table 7 presents data on the hydration status, fluid intake and heat index during training for each of the seven teams. The mean temperature and relative humidity was 20.1°C (10.2 to 34.1°C) and 58% (25 to 94%) respectively. Although the mean heat index was < 26.7°C for the majority of training sessions, teams one (temperature range: 22.2 to 34.1°C; RH range: 25 to 78%) and seven (temperature range: 13.4 to 18.4°C; RH range: 32 to 71%) trained under conditions where the heat index (26.7 to 31.7°C) during both training sessions placed them at risk of possible fatigue with continued exposure and physical activity [23].

**Table 7:** **Hydration status, fluid intake and heat index for each team**

**Training session one**								**Training session two**							
**U**_**SG**_ **before training (g/ml)**	**U**_**SG**_ **after training (g/ml)**	**Hydration status before training**	**Hydration status after training**	**Percentage weight loss (%)**	**Amount of players consuming water n (%)**	**Water intake (ml)**	**Heat index (°C)**	**U**_**SG**_ **before training (g/ml)**	**U**_**SG**_ **after training (g/ml)**	**Hydration status before training**	**Hydration status after training**	**Percentage weight loss (%)**	**Amount of players consuming water n (%)**	**Water intake (ml)**	**Heat index (°C)**
**Team One (n = 8)**															
1.021 ± 0.005	1.019 ± 0.009	Slightly dehydrated	Euhydrated	0.9 ± 0.6	5 (63)	132 ± 96	28.8	1.024 ± 0.006	1.024 ± 0.007	Slightly dehydrated	Slightly dehydrated	0.7 ± 0.6	3 (38)	133 ± 142	28.2
**Team Two (n = 8)**															
1.024 ± 0.005	1.022 ± 0.009	Slightly dehydrated	Slightly dehydrated	0.5 ± 0.3	3 (38)	148 ± 98	26.6	1.025 ± 0.004	1.027 ± 0.002	Very dehydrated	Very dehydrated	0.8 ± 0.4	0 (0)	0.0 ± 0.0	26.6
**Team Three (n = 15)**															
1.025 ± 0.004	1.025 ± 0.006	Very dehydrated	Very dehydrated	0.6 ± 0.4	14 (93)	229 ± 45.5	23.2	1.020 ± 0.005	1.021 ± 0.006	Euhydrated	Slightly dehydrated	0.3 ± 0.7	15 (100)	340 ± 140.	23.8
**Team Four (n = 8)**															
1.025 ± 0.002	1.023 ± 0.003	Very dehydrated	Slightly dehydrated	0.9 ± 0.1	1 (13)	252.6 ± 253	22.5	1.019 ± 0.007	1.020 ± 0.008	Euhydrated	Euhydrated	0.9 ± 1.2	1 (13)	22 ± 22	23.4
**Team Five (n = 12)**															
1.020 ± 0.008	1.022 ± 0.007	Euhydrated	Slightly dehydrated	0.5 ± 0.7	6 (50)	151 ± 93	25.6	1.025 ± 0.006	1.026 ± 0.005	Very dehydrated	Very dehydrated	0.8 ± 0.5	4 (33)	60 ± 70	31.4
**Team Six (n = 14)**															
1.024 ± 0.006	1.023 ± 0.007	Slightly dehydrated	Slightly dehydrated	0.5 ± 0.4	3 (21)	167 ± 124	30.3	1.024 ± 0.005	1.024 ± 0.007	Slightly dehydrated	Slightly dehydrated	0.6 ± 0.4	0 (0)	0.0 ± 0.0	26.0
**Team Seven (n = 14)**															
1.024 ± 0.006	1.025 ± 0.005	Slightly dehydrated	Very dehydrated	0.9 ± 0.4	1 (7)	34 ± 34	26.9	1.026 ± 0.005	1.026 ± 0.005	Very dehydrated	Very dehydrated	1.0 ± 0.5	0 (0)	0.0 ± 0.0	27.6

Team one who played for seventy minutes did not seem to be affected as they remained slightly dehydrated before and after both training sessions although one player (1/8) in the first session and three (3/8) in the second session became extremely dehydrated. Team seven who only played for 40 minutes, tended to be very dehydrated (U_SG_ 1.025 to 1.026 g/ml) before and after both training sessions, lost the largest percentage body weight (0.9 to1.0%) and consumed no fluids.

More than half of the players (57%, 45/79) did not consume any fluid during the first training session. The mean total fluid intake (water and pure fruit juice) during the first training session was 181 ml (3 to 269 ml). Pure fruit juice was the only source of carbohydrate for the players as none consumed any food during training. The mean water intake (33/79) was 183 ml (21 to 269 ml) and the mean pure fruit juice intake (4/79) was 31 ml (10 to 57 ml). The majority (71%, 56/79) did not consume any fluid during the second training session. The mean total fluid intake (23/79) was 250 ml (12 to 506 ml). The mean water intake (23/79) during the second training session was 250 ml (12 to 506 ml) while no players consumed any pure fruit juice.

Less than 50% of players in all teams consumed fluid during training. The exception was team three where nearly all players consumed fluid during both sessions. Teams two, six and seven did not consume any fluid during the second training session.

### Knowledge regarding the importance of fluid and carbohydrate

#### Fluid

Eighty one percent (64/79) of players completed the self-administered questionnaire. The majority (>90.0%) stated that water was the most important fluid to consume before, during and after training. Energy drinks (6 to 8% carbohydrate solutions) were considered important for before training by 31%, during training by 21% and after training by 16% of players. Similarly, non-carbonated cold drinks (such as fruit cordials which are 4 to 8% carbohydrate solutions), were considered to be important before training by 29%, during training by 14% and after training by 16%. All other carbohydrate containing beverages such as pure fruit juice, recovery drinks, high energy drinks and carbonated drinks were not considered to be important by the majority of players. Some players stated that fluid is not important before training (30%), during training (17%) and after training (7%).

The majority of players stated that 500 to 1000 ml of fluid should be consumed before (46%), during (35%) and after training (71%), amounting to a total of 1500 to 3000 ml per training session. Others (32%) believed that 200 to 500 ml of fluid should be consumed before training, 0 to 200 ml (26%) during training and 200 to 500 ml (10%) after training.

### Carbohydrate

The majority (74%) stated that carbohydrate was important for soccer training, although some (40%) believed that carbohydrate should only be consumed before training. None of the players stated that carbohydrate should be consumed during training. However, a few (5%) stated that carbohydrate should be consumed before, during and after training.

## Discussion

The finding that 28% of the players were severely wasted was not surprising, considering the high rate of unemployment, poverty and food insecurity among black South Africans [17,18,26-29]. As wasting is one indicator of acute malnutrition, it is possible that these players did not have access to sufficient food for optimal growth, let alone that required to supply additional energy and carbohydrate required for soccer training [26].

Similarly to previous studies conducted on adolescent soccer players during training, the players on average, were slightly dehydrated before and after training [19,30]. Therefore, players arrived for training in a less than optimal hydration state and were not addressing this during training by hydrating adequately. Although players did not appear to be at risk for significant dehydration, over a fifth were in fact extremely dehydrated before and after training identifying a high risk population that needs further investigation.

The lack of facilities such as taps at training grounds, inadequate fluid intake and training at a high heat index (26.7°C to 31.7°C) put specific teams at risk of dehydration. This was evident in team seven who in spite of only training for forty minutes, lost the largest percentage of body mass (0.9 to 1.0%) and tended to be very dehydrated both before and after training.

The degree of dehydration after training was acceptable as it was less than 1% [31]. This finding is similar to previous studies conducted among adolescent soccer players during training which found that players dehydrated at a level of 0.5% to 1% [19,30]. However, adult soccer players dehydrated at a level of 1.6% to 3.4% [32-35]. Male adolescent soccer players were therefore less dehydrated than their adult counterparts, possibly due to physiological and metabolic differences as adolescents do not sweat as effectively [4]. Relatively few players incurred levels of dehydration (≥2%) that were significant enough to impair performance and impact on health [2]. However, a less effective sweating mechanism predisposes adolescents to a greater risk of developing heat illness, thereby underlining the importance of appropriate hydration practices even when the percent loss of body mass does not appear of consequence [4,36].

Conversely, a few players (<10%) were extremely hyperhydrated before and after training. Hyperhydration has previously been documented in adult soccer players [30]. Excessive consumption of water results in hyperhydration which dilutes the extracellular concentration of sodium to levels that can have negative effects on the body [37]. An education program aimed at the players in this study would need to emphasize the risks involved in the overenthusiastic consumption of fluids, particularly water.

The majority of players in all teams (TS1 57% and TS2 71%) did not consume any fluids during the first and second training sessions. This is a concern as it is well established that fluid intake during training is important for athletes [38]. Players in teams two, six and seven did not consume any fluid during the second training session, possibly because these teams did not have a tap in the vicinity of their training grounds and may have forgotten to bring fluid. The majority of players in team three consumed water during training. This is possibly because there was a tap in the vicinity of the training ground and the majority of players from this team brought bottles to training. The main source of fluid was water which is in agreement with previous studies conducted on adult and adolescent soccer players [2,19,33,34]. None of the teams were provided with any fluids. Although not optimal for rehydration purposes, consuming water is better than consuming no fluid at all and is appropriate in cool weather conditions during training of moderate intensity [39,40]. Although more than 90% of players stated that water was the most important fluid to consume before, during and after training, only 42% (TS1) and 29% (TS2) actually consumed water during training. Thereby illustrating that their beliefs and actions were contradictory. Although the majority thought that water was the most important beverage to consume before, during and after training regardless of the duration of the training session, the majority did not consume this in the amounts that they believed to be appropriate.

Although most players stated that 500 to 1000 ml should be consumed during training, the mean consumption was 216 ml which is less than the recommended 150 to 200 ml of fluid every 15 minutes [41]. Fluid consumption during training is important to ensure the replacement of fluid lost through sweat and reduce the level of dehydration incurred [2,7]. The mean fluid consumption documented during training was substantially lower than that previously reported, as findings were that on average, adolescent soccer players consumed a total of 1133 ml of water during regular scheduled drink breaks as well as during actual training sessions [19]. The low fluid intake documented in the current study could be attributed to the lack of regular scheduled drink breaks, some players did not bring drink bottles to training, the lack of taps at the training grounds as well as insufficient knowledge regarding the importance of hydration by both the players and coaches. Despite consuming less fluid than reported by previous studies, players in the current study were less dehydrated possibly because they trained at a lower temperature for shorter periods of time [19].

The players did not have knowledge regarding adequate amounts or types of fluid to consume to prevent dehydration and supply adequate carbohydrate. This lack of knowledge can be detrimental to health as simple dietary measures such as consuming the correct type (6 to 8% carbohydrate beverages) and amount of fluid can protect health and maximise performance [42]. It is possible that this lack of knowledge was the result of a lack of exposure to nutrition education materials and interventions whether they are formalized or are as a result of exposure to mass media programmes.

The only source of carbohydrate consumed during training was very limited amounts of fruit juice consumed by some players as none consumed food while at the training ground although the majority claimed the carbohydrate was important for training. However, this did not correspond with their beliefs as few players believed that pure fruit juice was important. Pure fruit juice has a carbohydrate content of greater than > 10% which exceeds the recommended concentration of 6 to 8% and could therefore reduce gastric emptying resulting in decreased carbohydrate absorption [38].

Although carbohydrate consumption is not essential for training sessions with a duration of less than 60 minutes (such as the ones that took place during Winter), carbohydrate was important for the longer training sessions (70 minutes) in Autumn to maintain blood glucose levels and sustain athletic performance [38,43,44]. Although in theory the intake of carbohydrate is not as important for exercise of short duration players from a low socio-economic environment, such as these where 28% of players were classified as wasted may well need to consume carbohydrate during training. Food insecurity may imply that these players were not able to adequately replenish glycogen stores between training sessions and therefore maintain blood glucose levels. Although unable to afford the 6 to 8% carbohydrate electrolyte beverages, these players could be encouraged to consume fruit cordials with a carbohydrate content of 6 to 8%. A local drink made from fermented porridge (amahewu) would be suitable to consume before and after training. These are inexpensive, culturally acceptable and would contribute to hydration goals. Fluids that are flavoured have been found to increase fluid intake in young athletes, thereby reducing dehydration [5,12]. It is important to educate players to consume inexpensive foods and drinks before and after training to ensure that they arrive at training euhydrated with replenished energy stores. Inexpensive culturally acceptable foods would include both fermented porridge and fermented milk (amasi).

## Conclusion

The players’ background of poverty and severe wasting documented in the current study suggests that some adolescent soccer players may not have had access to sufficient food for adequate growth, let alone the nutrient intake to meet the additional demands of playing soccer. This finding has not previously been documented in studies conducted among adolescent soccer players.

The environmental conditions and lack of facilities such as taps at training grounds placed some players at risk of heat illness. This underlines the need to campaign for improved training facilities and to provide nutrition education programmes for socio-economically disadvantaged soccer players in South Africa. High risk players and teams were identified that were very dehydrated although the majority were only slightly dehydrated and a few were hyperhydrated. These players need to be identified and educated.

Many players did not consume any fluids. Those who did preferred water which was consumed in inadequate amounts. The players’ practices corresponded with their beliefs as most believed that water was the most important fluid to consume and few believed that carbohydrate containing drinks offered any benefit. However, none consumed fluid in the volume that they stated was appropriate.

The majority were unaware of the amounts and types of fluid necessary to prevent both dehydration and hyperhydration. This illustrates the importance of nutrition education targeting players, coaches and parents on the role of carbohydrate and fluid for soccer training.

The uniqueness of this study was its focus on the hydration practices of socio-economically disadvantaged male adolescent soccer players during training as opposed to matches. This data could serve as a guideline for nutrition education programmes targeting socio-economically disadvantaged soccer players.

The limitations of the study included the classification of hydration status according to U_SG_ categories based on research conducted among adult athletes due to a lack of published data on adolescent athletes. As this was a pilot study player electrolyte losses were not determined. The fluid intake both pre and post training could not be measured which may limit the value of the data. The players’ fluid consumption could have changed because they were being monitored.

## Abbreviations

BMI: Body mass index
PADSA: Pietermaritzburg and district soccer association
WHO: World health organisation
U_SG_: Urine specific gravity
